# Medical Miscellanies

**Published:** 1817-11

**Authors:** 


### PART IV.

### MEDICAL MISCELLANIES.

### THE PORTE FEUILLE, No. IX.

OR,

DEPOT FOR DETACHED FACTS AND INTERESTING PHÆNOMENA OCCURRING IN THE PRACTICE OF THE FRIENDS OF THE MEDICO-CHIRURGICAL JOURNAL.

"Ore trahit quodcunque potest atque addit acervo."

*Two Cases of violent Cerebral Affection; one proving fatal.*

*John Wilson*, ætat. 27, belonging to his Majesty's sloop Surinam, in the West Indies, was found, early in the morning of the 25th of December, 1809, in his hammock, apparently insensible.

Upon attempting to rouse him, he immediately relapsed into a state of coma resembling sleep. He had been observed to be rather deranged the preceding evening, but went to bed at the usual hour, and was found in the morning as above described. He was of a full plethoric habit, the pulse regular. — Statim fiat venæsectio ad ℥xvj ; applicetur emplastrum lyttæ toto capiti raso.— ℞. Magnes sulp. ℥jß ; pulv. jalapæ ʒß ; inf. sennæ ℥iij ; fiat haustus statim sumendus. The draught was repeated in two hours ; after which he had three stools, but which passed involuntarily. He continued in the same comatose state until the next morning, when he became more sensible, but he still continued speechless. At ten, A. M. he was attacked with a general spasmodic affection of the whole body, the muscles of the face being particularly distorted. It continued about two minutes, and returned thrice in the space of an hour. A bolus of camphor, gr. x, and opium, gr. j, was given every four hours. He had frequent stools during the day, and continued perfectly tranquil until the evening, when he had another fit, which recurred three times during the night, and at seven next morning he expired, forty-eight hours after he was first found in a comatose state, without having spoken a word. Permission was not obtained to open the body.

CASE 2. Frederick Pitchger, seaman, aged 29, in the evening of the 10th of January, 1810, was observed running about the deck in a state of derangement, and was immediately secured. He was, when visited, in a state of furious delirium, with a quick full pulse : Mittantur sanguinis e brachio, pleno rivo, unciæ viginti, et capiat haustum purgantem, ut supra prescriptum ; necnon injiciatur enema catharticum. From excessive struggling, he lost about five ounces of blood more than the quantity ordered, which produced syncope. The bowels were well opened during the night, and he became more tranquil. When visited, in the morning, he was lying in a comatose state, pulse regular ; abradatur capillitium et applicetur toto capiti emplastrum lyttæ. He contiuued in the same state for twelve hours, when he became perfectly sensible, and complained much of strangury from the blister, which was removed and dressed. He continued quite sensible, and progressively amended until the 26th of January, when he was discharged to duty.*

### *Case of Rupture of the Aorta (Sept.* 1809*).*

A man, belonging to the Ethalion frigate, was found dead in the morning. On opening the body, the abdominal cavity was distended with coagulated blood, and the aorta was discovered to be ruptured about an inch below the diaphragm. The coats were in a complete state of ossification ; the viscera were sound.

* These Cases are introduced to shew the danger of not using copious evacuations early. ED.

*Extract of a Letter from* R. CROFTON, *Esq. of Rutland, in Carlow, Ireland, dated Sept.* 20, 1817.

"This country suffered dreadfully during last summer from poverty; and now we have its constant attendant, I mean the typhus fever, in almost every cottage: perhaps I should not have mentioned this, but to give me an opportunity of speaking of the effects of spirit of turpentine.

"In some of the cases which I attended, and where such torpidity of the bowels took place as purgatives of another kind, given in any way, could not remove, I was tempted to try it in a dose of from half an ounce to an ounce, mixed with castor oil and mucilage, or with the yolk of an egg. It always succeeded, and I wish you would make some of the young men of the hospital try it under your own eye.

"I was induced to use it, from seeing it some time ago recommended, in cases of puerperal fever, in a periodical Magazine, published in Dublin, by Dr. Brennan; he only excited, at the time, the ridicule of the Profession."

---

*Case of Hydrencephalus Acutus, in which Petechiæ appeared.* Communicated by a Physician.

*August* 8, 1817.—D. R. a boy, ætat. 12, of a delicate habit, complains of occasional griping pains in his belly, accompanied with head ach, and want of appetite; his eyes appear dull and heavy, and he seems to hang his head in a peculiar manner. According to report, he frequently picks his nose, and grinds his teeth during sleep. Pulse 80; tongue rather foul. Stools of a greenish colour; urine natural. Has had an emetic administered twice without relief.—Capiat, pulv. submur. hyd. gr. iv; et secundo quoque die repetatur medicamentum. Adhibeatur pediluvium vespere.

12th. Has been very restless during the night, frequently awaking with loud screams; complains much of head-ache, but pain of the abdomen gone. Pulse 100, weak; tongue foul; urine high-coloured. Has been delirious during the night; the retina seems morbidly sensible to the impression of light.—Appl. vesicator. capiti et emplast. sinapeos pedibus. Cont. submur. hyd. et habeat fructum recent. (anglicè gooseberries), ad libitum.

14th. There is little alteration in the symptoms. Pulse 120, weak; heat of surface about 100°. The application of a bright light to the eye seems to cause much uneasiness.—Caput iterum exulceretur cum vesicatorio. Omitt. submur. hyd. et capiat super-tart. potas. ℥ß, mane et vespere. Adhibeatur lavatio tepida sub. vesperem.

16th. There is an eruption of dark purple or bluish-coloured spots, of no determinate size, over the whole body, but more particularly numerous upon the arms, breast, and neck. The urine and stools pass off involuntarily: the delirium is succeeded by stupor. He seems to squint a little with the right eye; his breath is fœtid, and there is a cadaverous smell over the whole body. Pulse 96.—Capiat pulv. cinchon. off. ℥i, in. horis viginti quatuor, partitis vicibus. Habeat vini rubri cyathum quarta quaque hora.—Continuentur alia.

18th. Stupor and coma still continue; petechial eruption rather fading; pulse 130, intermittent; the pupil is insensible to the impression of light; fæces and urine passed involuntarily. Cont. medicamenta u. a.

21st. Died this morning.

*Dissection.* Upon removing the skull-cap and dura mater, the first thing worthy of notice that came under my observation was the turgid and inflammatory appearance of the blood-vessels of the brain, along with an effusion of a milky-looking fluid, resembling coagulable lymph, between the pia mater and surface of the brain. In cutting down upon the two lateral ventricles, I saw several of those hard strumous swellings, or knots, which have often been taken notice of by authors, in treatises concerning hydrencephalus. The two lateral ventricles were distended with serum, as was also the case with the fourth ventricle. The quantity of water was not measured, but might probably amount to a number of ounces.

These are the chief of the morbid phænomena that came under my observation, with the exception of a very singular appearance in the sella turcica; namely, the total disappearance of the pituitary gland: and, in its place, a quantity of purulent matter. Petecchiæ are a frequent occurrence in hydrencephalus acutus.

D. C.

October 19, 1817.

---

### MISCELLANIES.

Quicquid agunt Homines.

---

### *To the Editors of the Medico-Chirurgical Journal & Review.*

GENTLEMEN,

IN the September Number of your valuable and justly-esteemed Journal, containing a list of those on whom the University of Edinburgh has lately conferred the degree of M. D. it gave me peculiar pleasure to find the names of so many medical officers from both branches of the public service. Nor is it only on the late occasion that Gentlemen of the above Class have sought the honours of Medicine in that distinguished School; I have reason to know, that for the three immediately preceding years there has regularly been a number very little inferior to the present, particularly of Naval Surgeons; and that, at this moment, several more are treading the same creditable course, for the graduation-day of the ensuing year.

That these officers, while but just emancipated from the fatigues of the campaign or the cruize, where haply they had been "knocked up in their shirt" to attend the duties of their station, or had, perchance, pined for months—nay years, under the insalutary influence of service in foreign climes, should occupy the first months of their retirement (when repose is so especially desirable) in receiving and adding to their medical knowledge, and in subjecting themselves to Examinations and Academic Exercises, which are tests of a man's *literature* as well as of his *professional* proficiency, is certainly a circumstance worthy of being noticed to their honour. They have not suffered their minds to be exposed to the debilitating atmosphere of idleness, or their talents to rust in vapid inaction, but seem to be animated by a spirit which tells them—and tells them truly—that their professional acquirements are an honourable charge, which they hold in trust for the benefit of their country; a *perishable* charge too, which they are bound to hold in perpetual *repair* and readiness for her service.

Did the circumstance I have just mentioned redound merely to the honour of the individuals alluded to, I should go no farther beyond paying them the above compliment, as a tribute due to their diligence and ardour for Medical Science, and would bid them adieu with every complacent wish for their future welfare. But I contend that it has an additional interest, inasmuch as it is a circumstance from which Patriotism may extract a spark of pleasure in the retrospect, and Philanthropy obtain an *ex-post-facto* consolation for some of the woes of war: for surely the lover of his country and of mankind, will be gratified to infer from it, that those "Great Hearts," who so gallantly fought, and so devotedly bled in our defence, during the season of protracted warfare, did not, while throbbing under the paroxysm of disease, or palpitating and fainting under the anguish of wounds, fall under the medical management of men of mean or secondary capacity. I am not aware, that the competency of those entrusted with the health of our brave defenders, have ever been seriously questioned by men whose opinion is of the least weight or value on such a point; but if it has, I would *now* ask, in what have they shewn themselves behind-hand?

Before taking leave, I beg to be indulged with a remark or two on a circumstance of a more unpleasant description, which the tenor of this letter has brought to my recollection: I mean, the galling disparity in the remuneration granted to the medical officers of the Navy, compared with those of the Army. The former are not only obliged to solace themselves in their retirement with a stipend very considerably inferior to that of their brethren in the Army, but the same unjust, because partial, spirit pursues them with parsimonious rigour even beyond the grave, and visits upon their helpless widows a corresponding inequality of privilege in regard to pensions.

Dr. TROTTER long ago advocated the claims of Naval Medical men, and pronounced them a "a neglectum genus." Though much has been done since his time to the amendment of their situation, still some marks of their ancient *Helotism* yet remain; of which marks, the inequality here complained of, is at once the most unjust, the most degrading, and the most offensive. I am very far from thinking that the medical officers of the Army have too much:—most assuredly they have not. But I hold that those of the Navy ought to have *as much.* The remuneration granted to the sister services, ought to be on a scale of entire equality; since it cannot be maintained, with a shadow of truth or candour, that the science of the *Cock-pit* has proved itself a jot inferior to that of the *Camp.*

The medical officers of the Navy have a right to look for an equal reward from their country:—If they have not hitherto obtained, they have at least hitherto deserved it!

I am, &c.

VERIDICUS.

*September the 29th,* 1817.

*We are unwilling to admit any thing into our work which does not strictly relate to medical science or intelligence; yet (though the concluding part of* VERIDICUS's *letter bears no very direct reference to that object,) we should be sorry to shut our pages against the advocate of an enlightened and meritorious body of men, for whom, without any narrow or exclusive partiality, we feel very sincere respect.*

EDITORS.

---

### *On the Laws of Muscular Motion.* By J. R. PARK, M. B. &c.

(From the Journal of Science.)

MOTION is performed by means of muscular fibres; and all the functions of the body will be found to depend, either directly or indirectly, upon muscular contraction; the phænomena of which, when carefully examined, will also lead us to the knowledge of some general principles.

As sensation, when subservient to what are called animal functions, is attended with conciousness in the mind, as in the impressions made upon our organs of sense; so motion, in the same class of functions, is accompanied or preceded by an effort of the will, as in the voluntary exertion of our limbs.

But on the other hand, as sensation in automatic or organic functions is unattended by mental perception; this being the case with impressions on internal organs: so muscular motion, in this class of functions, is performed without any act of volition; as in the actions of the heart, stomach, and intestines.

Voluntary and involuntary motion present phænomena so different, as to have led some eminent physiologists to regard them as distinct faculties, radically different in their nature. By a separate consideration of each, it will the more readily be shewn, what ground there is for this conclusion.

Voluntary motion is allowed by all to consist in an altered condition of the moving fibre, produced through the immediate influence of the brain and connecting nerves.

The nature of the change effected is a longitudinal approximation of the particles composing the fibre; producing a consequent diminution of its length, with some little increase of its thickness.

That very little change of bulk however attends, has been proved experimentally, by immersing the arm in water, previously to contraction, and then throwing the muscles into action; which caused no perceptible change in the height of the fluid, though contained in a vessel with a graduated tube affixed to it, in such a manner as to have detected the slightest variation of bulk in its contents.

The means or agent that causes this change of condition in the moving fibre, is, however, the point which has most engaged the attention of physiologists, when it might perhaps have been more profitably employed in considering the effects.

The laws of muscular motion, like those of matter, must be deduced from the phænomena. To these then we shall now turn our attention.

The most familiar and remarkable circumstance attending voluntary motion, is the progressive change which the powers of action undergo from continued exertion.

When we begin to move our limbs, we usually find the first efforts are comparatively feeble and inert. But as we continue our exertions, until we grow warm with action, the energies of the body are gradually developed, and we soon attain to the full possession of our strength and activity. After a further period of exertion, this state of vigour and activity is succeeded by a sense of uneasiness, which we call fatigue; and eventually we are compelled to desist from our efforts by the painful sensation they occasion, or the want of power to continue them.

These changes, though subject to considerable variety, in regard to degree and duration, may yet be perceptibly traced through all voluntary organs; and from these we may venture, without any knowledge of the cause that produces them, to lay down the following as laws of muscular motion.

1. That exertion is productive of certain changes in the organs of motion, which are accompanied by corresponding changes in the powers of action.

2. That the powers of action have certain limits prescribed to them, which sooner or later require the renovation derived from rest.

The progressive changes which the powers of action undergo, from continued exertion, vary in almost every organ; and are subject to farther variation from the influence of habit; but for the sake of precision, they may be distinguished into three stages.

First,—The stage of inertia, or comparatively inefficient activity.

Secondly,—The stage of vigour, or the period of energetic action.

Thirdly,—The stage of fatigue, or painful and difficult exertion.

The degree of rest that is required to restore the powers of action, also varies according to the nature of the organ, the previous degree of exertion, and the influence of habit. But however rapid the renewal of power in some parts, or however protracted the period of exertion in others, still all voluntary organs manifestly have certain limits, beyond which, the continuance of action becomes impossible, and rest indispensable.

Although the operation of these laws at present is restricted to voluntary organs, yet it will hereafter be seen, that they equally apply to those which are involuntary, and really admit of no exception. And this universality of the law certainly affords a presumptive proof that these alternations of action and rest proceed from some physical necessity, some change of condition in the moving organ, which is inseparable from muscular contraction.

Without inquiring into the nature of the nervous influence, we may readily conceive the probability of its producing such changes as those in question.

If, as some suppose, it be at all analogous to the Galvanic energy in its mode of operation, it seems in no wise improbable, that its first application should not produce their full effect on the muscular fibre ; and therefore that the powers of action should not at once be fully developed.

That the fibre should gradually become more susceptible of its influence, as we grow warm with action, seems also every way probable, and thus we should expect that the powers of action would for a time increase with exertion.

Further, that the repeated application of so powerful an agent, should at length produce changes that are excessive and painful, is analogous to what occurs from all other changes that are too rapid in their production, or too considerable in degree.

The opinion contended for, is this : that the sensation of fatigue arises from a change of condition in the moving fibre, effected by the repeated application of the nervous influence. Of this change, no matter what be its nature or cause, the mind becomes conscious when it arises at a certain degree, because the muscular structure, like every other, has some portion of nerve blended with its substance, and entering into its composition.

The opinion to be combated is this: that the sensation of fatigue arises from the expenditure or exhaustion of that power or energy imparted by the nerve to the moving fibre ; and not from the changes produced by its repeated application.

If not an offspring of the Brunonian hypothesis, this doctrine is so nearly allied to it, that a brief outline of that system is necessary to place it in a clearer point of view.

The system alluded to, was about the same period advanced by Brown and Darwin ; and soon obtained disciples, by its apparent simplicity, which promised to save the trouble of any further research, and explain, in a summary way, all the mysteries of our nature.

We were only required to admit, that the brain is capable of secreting or generating a certain energy, termed by Dr. Brown, excitability; and by Dr. Darwin, sensorial power: and allow this to undergo various degrees of accumulation and expenditure; and its different states of fluctuation were deemed adequate to explain all the leading phænomena of life.

A deficient expenditure of this power was conceived to occasion *direct debility*, by allowing a morbid accumulation.

An excessive expenditure was said to cause *indirect debility*, by producing an exhaustion of this power.

Every impression acting upon the mind or body, and every action exerted by it, were conceived to occasion a loss or expenditure of excitability; fatigue was, therefore, a state of indirect debility, proceeding from immoderate expenditure of sensorial power.

In the first place,—The assumption on which this rests, is gratuitous and unfounded.

Secondly,—The phænomena are incompatible with this supposed cause, if its existence were proved.—And

Lastly,—They all perfectly accord with the view already offered, of a change in the moving fibre itself; which, moreover, may be directly proved to take place.

To return.—The assumption that the nervous energy is liable to be exhausted in the living body, appears unfounded.

This exhaustion, if it occur, must be either partial or general. We will first suppose it partial.

Whatever be the nature of this fluid, power or energy, rapid diffusibility is one of its most indisputable properties. The experiments of Dr. Wollaston, related in the Croonian Lecture for 1810, which render it probable that every muscular contraction consists of a number of separate shocks, following each other in such rapid succession, as to appear only a single effort of contraction, may convey some idea of the amazing velocity with which this energy is imparted to the nerve by the muscular fibre.

From this rapid diffusibility then, it would be expected, that the effects of a partial expenditure should be no longer felt than the time required for the exhaustion in one part to be replenished from others, or from the general reservoir, the sensorium, according to the Brunonian doctrine.

How is it then, that the pain of fatigue, and the effects of over exertion, often last for many days, or even weeks? Is this power so slowly diffused, or is it so long in re-accumulating?

But the exhaustion of excitability may be general, which we will next suppose it to be.

If the cause be general, the effects should be so likewise, and the over exertion of one limb or organ should cause the sense of fatigue to be felt in others, or in all. This, however, is so far from being the case, that an extraordinary exertion may incapacitate one arm for several days, and the rest of the body feel no participation.

During the period of exertion, when the effort begins to excite painful sensations, merely changing the mode of action is often sufficient to alleviate weariness, even in the same limb, by calling other fibres into action, and allowing those to rest which were previously exerted.

Now, the pain would not be thus removed, by merely directing the nervous energy into a fresh channel, if its expenditure, and not its application, were the exciting cause of that pain.

Thus, there seems no ground for supposing such an expenditure of the nervous energy to take place, as to occasion pain from either a partial or a general exhaustion.

But, secondly, were such an exhaustion allowed to take place, the sense of fatigue and the phænomena of muscular action, are not reconcilable with this, if assumed as their cause.

If the powers of action depend solely upon the due accumulation of this energy, and at length are suspended, in consequence of its exhaustion, then the activity should uniformly decline as the power is expended, and finally cease altogether when the period of exhaustion arrives.

Instead, however, of this being the case, activity is usually found to increase with exertion. Thus, the first dance is generally the most fatiguing; and a judicious sportsman spares his horse till he grows warm with action.

Further, when the powers of action appear nearly exhausted, they are often suddenly renewed by the very means, which, according to the Brunonian doctrine, should cause a still greater exhaustion; namely, a strong impression on the mind. Thus, a soldier sinking under the fatigue of a long march, when roused by the unexpected appearance of an enemy, awakened by the sense of danger, or animated by the hope of glory, feels his strength suddenly recruited, and forgets his fatigue.

Now, as every impression on the mind causes a further expenditure of excitability, according to the Brunonian system; this, instead of restoring the strength, should further augment the indirect debility which already prevails; and thus the phænomena of fatigue are incompatible with this exhaustion as their assumed cause.

Lastly, it remains to be shewn, that they are consistent with the conclusion, that fatigue is a sensation arising from a change in the condition of the moving fibre itself, effected by the continued application of the nervous energy, and that direct proof of the existence of this change may be offered.

The state of the moving organ, on the approach of fatigue, appears to be in fact that of over-contraction, and not that of relaxation in the muscles; and strong impressions on the mind, having a tendency to take off spasm, as proved by daily experience, and in a manner that will hereafter be explained, this immediately moderates the spastic state of the muscle, and restores its powers of action; just as friction, cordials, or a warm bath, would equally produce the same effect in cases of over-fatigue.

The efficacy of such remedies, applied to the moving organs, under similar circumstances, further attests that they are the immediate seat of the change that takes place; were this proof wanting to establish the fact, that a spasmodic state of the muscles is the real cause of fatigue. Of this, however, we have still more direct evidence in the actual condition of the organs themselves.

As fatigue approaches, nothing is more common than cramps or spasms in the calves of the legs, and the soles of the feet; denoting an excess, and not a want of contractility; therefore not a deficient, but an excessive application of nervous influence.

And when at length, exertion has been so long continued, that the power of motion is impeded, or supported with pain and difficulty, the condition of the muscles presents nothing like a state of relaxation, as if wanting the power to contract, but, on the contrary, a state of rigid firmness, wanting the power to relax. Both flexor and extensor muscles will be found at this period to be alike rigidly contracted, by which the limb is for a time immovably fixed, and the power of motion impeded, until this spastic state is removed, by means similar to those already mentioned; or until the contraction spontaneously subsides by rest and sleep.

Finally, we have the most conclusive evidence of the muscles being the seat of the changes that occur, in the sensible and visible appearance of those organs which succeeds to immoderate exertion. When the spastic state goes off, and is followed by a subsequent relaxation, which will be found always to bear a relation to the degree of previous over action, what is now the condition of the limbs?

It is one which could not have been disregarded, were physiologists more disposed to reason from obvious facts, which experience presents, and less attached to fanciful hypotheses.

The state of the muscle is one approaching to actual inflammation; in fact, a manifest change in the condition of its capillary vessels, attended with pain, heat, redness, and swelling; a condition requiring often the application of leeches, cupping glasses, and other modes of local evacuation, to unload the distended and weakened vessels, previously to the employment of those means which are best calculated to restore their natural tone.

( To be concluded in our next Number. )

---

### *History and Treatment of Endemic Fever in the Achates. In a Report to Dr. Dickson, then Physician to the Fleet in the West Indies.*

On the 20th of August, 1809, H. M. Brig, Achates, arrived perfectly healthy at English Harbour, Antigua; and though there only five days, yet from the people being employed in landing cordage, in hard work, and especially from exposure to intemperance, fever was induced, and cases continued to occur from day-to-day, during the passage to Barbadoes, till the 1st of September. The symptoms were, a severe pain across the forehead, and orbits; in the back and loins stretching towards the umbilicus; and, in general, a fixed pain at the præcordia, in the large joints, gastrocnemii muscles; with anorexy, nausea, vomiting, lassitude, and a sense of general debility. During the first hour or two the skin was cool; and in some cases there was a rigor, but more commonly alternate chills and flushes of heat; the pulse small, quick, and contracted; tongue clean; thirst; belly costive. After two or three hours the symptoms put on a very different appearance: the skin became hot and dry; the face flushed; the vessels of the tunica adnata turgid; the pain in the eye-balls and forehead increased, and, in some cases, delirium came on. The pain at the præcordia and irritability of stomach were now greatly increased; the pulse became hard and full, and varying in frequency from 100 to 120, and sometimes more: the patient became extremely restless, and was with difficulty kept in bed; others had a low degree of coma and a dread of death. If these symptoms were not arrested during the first two or three days, they were changed for others of a worse aspect (at least in those who died on board): the pulse became weak and small; the vomiting increased, and changed its appearance: the pains in the head, præcordia, &c. ceased; the eyes had a glistening appearance, and the patient sometimes asked for food; had free evacuations *per anum*, and expressed himself much better. A few hours before death, great debility, watchfulness, and low delirium supervened: the extremities became cold; hiccup; incessant vomiting; convulsions, and death.

These bad symptoms did not appear when the fever was arrested early; and the success with which this was attempted, was in proportion to the earliness of the evacuations, and the promptness in the application of the remedies.

Between the 20th of August and the 1st of September, when the vessel arrived at Barbadoes, about thirty men were seized with fever, three of whom died at sea. The sloop refitted in Carlisle Bay, and sailed in the middle of October, during which period, about a dozen men were attacked with fever or dysentery; and six were sent to the hospital, where two died of the former and one of the latter complaint.* The treatment, in general, consisted in the abstraction of ℥xviij of blood, the exhibition of a mercurial purgative, repeated if necessary; and, according to its

* This is one of the innumerable examples of the superior healthiness of Barbadoes, when compared with English Harbour, Antigua, or, in other words, of an open anchorage over a close and land-locked harbour. Had the Achates remained a few days longer at the latter place, there is not a doubt that the fever would have pervaded the whole crew, and would have been attended with considerable mortality. Edit.

effects, or as often as the skin became hot, *the cold affusion*, which generally produced a salutary perspiration and refreshing sleep. If the cathartic did not operate in five or six hours, an enema was given; and the sooner evacuations were procured *per anum*, the sooner was relief to the sufferer obtained, and the pain of the præcordia especially became much easier. If the pulse continued full and quick, and the head-ache, &c. were not relieved, the bleeding was repeated, and blisters applied to the head and epigastric region. In some, the application of rum to the head had a very cooling and beneficial effect in relieving the head-ache, and many expressed a wish to have it repeated. In the latter stage of the disease, from the recommendation of Dr. Cumming, late surgeon of the hospital at Antigua, the exhibition of calomel was tried to excite ptyalism, but I did not satisfy myself as to its bringing the fever to a more speedy termination; at this late period I gave a bolus of the carbonate of ammonia with camphor every hour, and in some cases it appeared to be of service. I have since thought I was too diffident in the practice of blood-letting, for future experience has fully convinced me of its great utility. I regretted much that I did not bleed the first man who died on board more freely in the first stage of the disease.*

---

*Extract from the late Report of the French Institute, on the Subject of Vaccination*, drawn up and sanctioned by the well-known and respected names of Messrs. Berthollet, Percy, and Halle.—"The preservative effect of the Vaccine Virus, when this virus has been taken in those determinate circumstances which assure us of its purity, and when the development of it has been complete, is, at least, as certain as that of the Small-pox itself, or as that which results from variolous inoculation. Moreover, considered with relation to Society in general, Vaccination has an advantage which Inoculation cannot possess, that of putting a stop to Variolous Epidemics, circumscribing them, and causing them to disappear; and of considerably diminishing the mortality which threatens the early periods of life—thus preserving to population its most advantageous proportion. In short, the results obtained, up to this time, inspire us with the probable hope of seeing the scourge of the small-pox, (one of the most deplorable under which human nature suffers) disappear from society."

---

A new metal has been discovered in the mines of Styria, the oxyds of which have the whiteness of salt; it has resisted a heat

---

* The *very early symptoms* which usher endemic fever of the West, have not been sufficiently attended to, and the foregoing description reflects great credit on the observation of Mr. Steel, the intelligent Surgeon of the ship. Editors.

of 150 degrees W, without being fused. Professor de Vest who discovered this metal, proposes to call it *junonium.*

The Lectures on the various branches of Medicine, in the University of Edinburgh, commenced on Wednesday the 29th of October, as follow:—Materia Medica, at eight o'clock, A. M. by Dr. Home; Practice of Physic, at nine o'clock, by Dr. Gregory; Chemistry and Chemical Pharmacy, at ten o'clock, by Dr. Hope; Institutions of Medicine, at eleven o'clock, by Dr. Duncan, sen.; Anatomy, Surgery and Pathology, at one o'clock, P.M.; by Dr. Monro; Natural History, at two o'clock, by Mr. Jamieson; Midwifery, at three o'clock, by Dr. Hamilton; Clinical Surgery, at five o'clock, by Mr. Russell.—Lectures on Clinical Medicine as usual. The introductory Lectures are delivered on the above day. After a fortnight's recess, the business of the respective Courses is commenced, and continued from the middle of November until the end of April.

The Annual Course of Lectures by W. T. Brande, Esq. Sec. R. S. Professor of Chemistry and Materia Medica to the Society of Apothecaries, will commence on Tuesday the 4th of November, at their Hall in Blackfriars, at two o'clock precisely, and will be continued on the third Tuesday in the said month of November, and on every first and third Tuesday in each month during the Season, at the same hour. The Lectures for the present season will relate to the Effects of Poisons, to their Detection, and to the Treatment of Persons poisoned.

### DEATHS.

At his house in St. Andrew's Square, Edinburgh, at the advanced age of 85, *Dr. Alexander Monro, Secundus,* Professor of Anatomy, Pathology, and Surgery in the University of Edinburgh.

In spite of professed levellers and all their coarse dogmas, and stern declamation about the native equality of mankind, and the emptiness of all adventitious distinctions, the mind is *unavoidably*, and therefore *naturally*, much more struck when one high in splendour and in place pays the debt of our common nature, than when the earth opens to receive one of her humbler children; just as we feel more powerfully agitated by the lightening of heaven striking the stately cypress, or the mountain pine; or splintering the cloud-girt spire of the populous city, (to which all eyes were wont to be lifted) than when it sears the shrub, blights the blushing rose, or lays in ruins the lowly cot in the lone vale.

The solemn impression of regret is more especially deepened when the individual, as in the present case, has been no less ennobled by the natural aristocracy of the virtues and talents, than by the imposing halo of rank and fame.

He is dead! the far-famed Physician and Pathologist, whose time was spent in prolonging life, or throwing light on the cause of death! In his decease, one of the brightest gems has fallen from the diadem of Medical Science.

To attempt a character of Dr. Monro would be superfluous, for in what country or clime are not his *Pupils* to be found? To have been one of these, the Writer of this Notice accounts a memorable as well as a fortunate circumstance in his medical education.

By all who heard them, the value of his Lectures will be long remembered. His eloquence was of that uncommon sort, that while apparently it aimed at nothing, it accomplished every thing. Perspicuous, impressive, and convincing, it had a touching simplicity and air of antiquity about it, which rendered it venerable; and completely exemplified what St. James, with singular felicity of expression, has called "the meekness of wisdom." From this eloquence, the driest and most uninteresting parts of his Anatomical Course caught a glow, and an interest, almost beyond belief. Even in the unpromising field of Osteology, he might be said, like the Prophet of old, to have infused animation into the *dry bones*.

It is curious to know, that this rare style was first evolved by an accident of rather a peculiar description. He had been in the habit of lecturing from Notes, but had one day forgotten them at home, and did not discover the omission till already in his Class-Room, and in the presence of his auditors. The situation was embarrassing; he decided on an attempt to go on without his manuscript, and succeeded so well, that he never afterwards used one!

Never did Physician deserve or enjoy, to a greater extent, the confidence of the Public. For accuracy of diagnosis in cases of organic lesions of the viscera, and of profound skill in the Pathology, more particularly of surgical diseases, he has very seldom been equalled, certainly never surpassed. His retirement from practice, and from the duties of his Chair, on account of the infirmities of age, about ten years ago, was regarded as a public loss. His demise may be considered *an event* in the History of Medicine. May those who heard his instructions with delight be so fortunate, now he is removed from us, as to catch a shred of his *mantle!*

"There is a tear for *all* that die,  
A mourner o'er the humblest grave:  
But Science heaves for *him* the sigh,  
A Nation's tears *his* urn shall lave!"

At Alford, Lincolnshire, aged 29, Mr. Thomas Allenby, Surgeon. — In Conduit Street, Hanover Square, aged 58, Mr. John Barclay, Surgeon. — Aged 27, Mr. Archibald Fraser, Assistant Surgeon to the 68th Light Infantry.

